# Changes in characteristics and case‐severity in patients hospitalised with influenza A (H1N1) pdm09 infection between two epidemic waves—England, 2009–2010

**DOI:** 10.1111/irv.12863

**Published:** 2021-05-04

**Authors:** Ian D. Plumb, Ross Harris, Helen K. Green, Joanna Ellis, Kathy Baisley, Richard G. Pebody

**Affiliations:** ^1^ Public Health England London UK; ^2^ London School of Hygiene and Tropical Medicine London UK

**Keywords:** comorbidity, hospitalisation, influenza A virus, pandemics, risk factors, socio‐economic factors

## Abstract

**Background:**

During 2009‐2010, pandemic influenza A (H1N1) pdm09 virus (pH1N1) infections in England occurred in two epidemic waves. Reasons for a reported increase in case‐severity during the second wave are unclear.

**Methods:**

We analysed hospital‐based surveillance for patients with pH1N1 infections in England during 2009‐2010 and linked national data sets to estimate ethnicity, socio‐economic status and death within 28 days of admission. We used multivariable logistic regression to assess whether changes in demographic, clinical and management characteristics of patients could explain an increase in ICU admission or death, and accounted for missing values using multiple imputation.

**Results:**

During the first wave, 54/960 (6%) hospitalised patients required intensive care and 21/960 (2%) died; during the second wave 143/1420 (10%) required intensive care and 55/1420 (4%) died. In a multivariable model, during the second wave patients were less likely to be from an ethnic minority (OR 0.33, 95% CI 0.26‐0.42), have an elevated deprivation score (OR 0.75, 95% CI 0.68‐0.83), have known comorbidity (OR 0.78, 95% CI 0.63‐0.97) or receive antiviral therapy ≤2 days before onset (OR 0.72, 95% CI 0.56‐0.92). Increased case‐severity during the second wave was not explained by changes in demographic, clinical or management characteristics.

**Conclusions:**

Monitoring changes in patient characteristics could help target interventions during multiple waves of COVID‐19 or a future influenza pandemic. To understand and respond to changes in case‐severity, surveillance is needed that includes additional factors such as admission thresholds and seasonal coinfections.

## 1 BACKGROUND

During 2009‐2010, influenza A (H1N1)pdm09 virus (pH1N1) infections occurred in two distinct epidemic waves in several countries, including in England^1^, ^2^, ^3^ and in the United States.^4^, ^5^, ^6^ Infections with pH1N1 leading to hospital admissions in England were reported to be more severe during the second wave than during the first wave,^3^, ^7^ in contrast to the United States^4^, ^5^ and Canada.^8^ A Bayesian analysis from available data estimated an increase in overall case‐severity in England,^9^ consistent with a sentinel surveillance study that noted patients hospitalised during the second wave were nearly twice as likely to be admitted to an intensive care unit (ICU) or to die following infection.^3^

However, it is unclear whether shifts in patient characteristics or health interventions between epidemic waves could have explained the reported increase in clinical severity. Sentinel surveillance from England indicated that patients during the second wave in England were generally older^3^; similar shifts were reported in the United States and Canada.^5^, ^8^ There was also a greater geographic dispersal of cases during the second wave, while fewer cases were reported from ethnic minority groups.^3^, ^5^ It is possible that this reflected differences in the socio‐economic status.^10^, ^11^, ^12^

Changes in health interventions might also have explained an increase in case‐severity. In England, there was a decline in the proportion of patients with pH1N1 receiving timely antiviral therapy during the second wave,^2^, ^3^ in contrast to the United States.^4^, ^5^ A tendency to admit only patients with more severe infections during the second wave might also explain an increased case‐severity among hospitalised patients.

Understanding whether increased case‐severity can be explained by changes in other patient characteristics could inform responses to COVID‐19 and to future influenza pandemics. We analysed national surveillance data of cases hospitalised during the 2009/2010 pH1N1 pandemic to determine whether reported increased severity of infections among hospitalised patients during the second wave could be explained by changes in reported patient characteristics or health interventions between waves.

## 2 METHODS

Surveillance data were collected for patients with laboratory‐confirmed influenza A (H1N1)pdm09 virus infection admitted between 27th May 2009 and 5th January 2010 to 129/166 participating NHS Trusts in England. Case information was collected using a standard form under the joint direction of the Health Protection Agency (HPA) and the Chief Medical Officer, as part of enhanced pandemic surveillance using methods reported previously.^1^

Case‐severity was defined as the probability that a hospitalised patient was admitted to ICU or died. Information on potential explanatory factors was gathered for case demographics (region, age, gender, ethnicity, underlying risk factors), and interventions (time to hospital admission, use of antivirals).

Further data sets were used to assess socio‐economic status, ethnicity, and mortality up to 28 days after admission. We used the Index of Multiple Deprivation (IMD) score to estimate socio‐economic status based on place of residence, with a higher score indicating more deprivation. This score is compiled by the Office of National Statistics (ONS), by lower super output areas (LSOAs) of approximately 300 residents which allow matching by a patient's residential postcode.^13^ We used quartiles of England LSOAs for the analysis. Ethnicity was estimated by matching to surname via an algorithm as previously described,^14^ summarising the results as either white British, or from an ethnic minority group. We matched cases to ONS death registration data for 2009‐2010 to prevent any bias through the censoring of late death registrations. We defined death as any death reported or matched in ONS data in the 28 days after admission to hospital.

Following the approach of an earlier analysis of the two waves of hospitalisations,^1^ we defined wave 1 as hospital admission between 27th May 2009 and 29th August 2009, and wave 2 as admission between 30th August and 3rd January 2010. We defined “any comorbidity” as respiratory, renal, neurological, cardiovascular or hepatic disease which could impair organ function; or reported diagnosis of diabetes, obesity, or immunocompromised status. We also reported pregnancy status. To assess the timeliness of admission we excluded cases with onset reported after admission. Since antiviral treatment is most effective within 48 hours,^15^ we categorised antiviral use as within 2 days of symptom onset, or later. For analysis of the association between antiviral use and ICU admission, we excluded patients who received antivirals after admission to ICU.

To account for missing values, in the primary analysis, we assumed that unrecorded ICU admission or comorbidity implied the factor was absent since the data were collected alongside routine clinical care; for other variables, we excluded missing values. To address potential bias from missing data, we conducted a secondary sensitivity analysis using multiple imputation, including for ICU admission and comorbidity. This method uses the distribution of the observed data to predict a set of credible values for the missing data.^16^ We used 20 imputed data sets, which is generally considered to be adequate^17^ and predicted missing values for the outcomes and exposures of interest in the primary model. If predicting variables were highly correlated with each other we used the most pertinent variable for imputation.

After comparing characteristics and case‐severity between epidemic waves for all hospitalised patients, patients admitted to ICU, and patients who died in the 28 days after admission, we used logistic regression to assess which characteristics were independently associated with epidemic wave. We used the hierarchical approach proposed by Victora and others to select variables to include in multivariable models,^18^ adjusting first for factors such as age, gender and region that we considered distal to the outcomes of interest. We then adjusted for comorbidities, followed by proximal management factors—timing of admission and antiviral use.

To identify potential explanatory factors for differences between epidemic waves, we modelled predictors of ICU admission and death. We then reviewed whether admission during wave 2 predicted increased case‐severity after adjusting for potential explanatory factors. The study was performed in accordance with the NHS Act 2006 (Section 251). Data were analysed using Stata 13.1 (StataCorp).

## 3 RESULTS

Of 2380 hospitalised patients included in the analysis, 40.3% (960 patients) were admitted during wave 1, and 59.7% (1420 patients) during wave 2; 36 patients were excluded because of unknown admission date. Whereas 60% of patients (n = 576) were admitted to hospitals in London or the West Midlands during wave 1, only 20.1% of patients (n = 285) were admitted in these regions during wave 2. Overall 66.5% of patients (1404/2110), patients were resident in areas with higher deprivation scores than the median level for England, including 81.2% of patients during wave 1 and 57.1% during wave 2. Similarly, the 28.7% of patients (684/2380) estimated to come from ethnic minority groups represented 47.6% of patients during wave 1 compared with 16.0% of patients during wave 2.

Overall, 41.2% patients (n = 981) had an identified comorbidity, including 44.4% (427/960 patients) admitted during wave 1 and 39.0% (554/1420 patients) admitted during wave 2 (Table 1). Proportions of patients with specific comorbidities also varied between waves (Table 1). From wave 1 to wave 2 there were declines in the proportions of patients admitted with asthma from 21.9% to 18.9%, with other respiratory disease from 7.2% to 6.5%, with neurologic disease from 6.8% to 3.9%, and with diabetes from 5.7% to 2.4%. By contrast, the proportion of patients with reported obesity increased from 1.7% (16 patients) during wave 1 to 2.8% (40 patients) during wave 2.

**TABLE 1:** Association between characteristics of hospitalised patients with influenza A(H1N1)pdm09 Virus infections and admission during wave 2—England, 2009‐2010

	No. patients with characteristic in wave 1/ all patients (%)^a^	No. patients with characteristic in wave 2/ all patients (%)^a^	Unadjusted odds ratio for admission during wave 2 (95% confidence Interval)^a^	Adjusted odds ratio for admission during wave 2 (95% confidence Interval)^a^
Region				
London/WM	576/960 (60%)	285/1420 (20%)	1	1
Other regions	384/960 (40%)	1135/1420 (80%)	5.97 (4.97‐7.17)	5.73 (4.61‐7.11)^b^
IMD quartiles^c^				
Least deprived	155/825 (19%)	551/1285 (43%)	‐	‐
Most deprived	670/825 (81%)	734/1285 (57%)	0.56 (0.52‐0.62)^c^	0.75 (0.68‐0.83)^b^, ^c^
Ethnic group				
White	503/960 (52%)	1193/1420 (84%)	1	1
Ethnic Minority	457/960 (48%)	227/1420 (16%)	0.21 (0.17‐0.25)	0.33 (0.26‐0.42)^b^
Age in years				
<15	390/960 (41%)	575/1420 (40%)	1	1
15‐44	403/960 (42%)	563/1420 (40%)	0.95 (0.79‐1.14)	0.89 (0.70‐1.12)^b^
≥45	167/960 (17%)	282/1420 (20%)	1.15 (0.91‐1.44)	0.94 (0.71‐1.25)^b^
Gender				
Female	503/959 (52%)	725/1416 (51%)	1	1
Male	456/959 (48%)	691/1416 (49%)	1.05 (0.89‐1.24)	1.10 (0.89‐1.35)^b^
Comorbidity				
Cardiac disease	46/960 (5%)	56/1420 (4%)	0.82 (0.55‐1.22)	1.33 (0.76‐2.32)^d^
Liver disease	7/960 (1%)	13/1420 (1%)	1.26 (0.50‐3.16)	1.95 (0.64‐5.1)^d^
Neurological disease	65/960 (7%)	59/1420 (4%)	0.60 (0.42‐0.86)	0.43 (0.27‐0.69)^d^
Renal disease	24/960 (3%)	39/1420 (3%)	1.10 (0.66‐1.84)	1.52 (0.79‐2.91)^d^
Asthma	210/960 (22%)	269/1420 (19%)	0.83 (0.68‐1.02)	0.73 (0.57‐0.95)^d^
Other respiratory disease	69/960 (7%)	92/1420 (6%)	0.89 (0.65‐1.24)	1.00 (0.65‐1.55)^d^
Diabetes	55/960 (6%)	34/1420 (2%)	0.40 (0.26‐0.62)	0.36 (0.20‐0.63)^d^
Immunocompromised	56/960 (6%)	86/1420 (6%)	1.04 (0.74‐1.47)	1.16 (0.74‐1.80)^d^
Obesity	16/960 (2%)	40/1420 (3%)	1.71 (0.95‐3.07)	1.64 (0.79‐3.40)^d^
Any specified comorbidity	427/960 (44%)	554/1420 (39%)	0.80 (0.68‐0.94)	0.78 (0.63‐0.97)^b^
Pregnancy	58/960 (6%)	83/1420 (6%)	0.97 (0.68‐1.36)	1.56 (0.97‐2.51)^d^
Onset to admission				
<2 d	461/862 (53%)	802/1380 (58%)	1	1
2‐4 d	298/862 (35%)	392/1380 (28%)	0.76 (0.63‐0.91)	0.66 (0.52‐0.85)^d^
>4 d	103/862 (12%)	186/1380 (13%)	1.04 (0.80‐1.36)	0.85 (0.61‐1.19)^d^
Antivirals ≤2 d of onset	307/730 (42%)	303/989 (31%)	0.62 (0.51‐0.76)	0.72 (0.56‐0.92)^d^

^a^ Wave 1: 27th May 2009‐29th August 2009; Wave 2: 30th August 2009‐3rd January 2010.

^b^ Adjusted for region, IMD quartile, ethnic group, age, sex.

^c^ IMD: Index of Multiple Deprivation Score, by quartile of scores for the population of England; odds ratios are for increased quartile.

^d^ Adjusted for region, IMD quartile, ethnic group, age, sex, neurological disease, diabetes, asthma.

The timing of admission and antiviral use also varied between waves. Although the proportion of patients admitted within the 2 days after symptom onset increased from 53.5% (461 patients) in wave 1 to 58.1% (802 patients) in wave 2, there was also an increase in the proportion of patients admitted 4 or more days after illness onset, from 11.9% (103 patients) to 13.5% (186 patients). Meanwhile, the proportion of patients receiving antiviral therapy within the 2 days after symptom onset decreased from 42.1% (307 patients) in wave 1 to 31.3% (303 patients) in wave 2.

Univariable and multivariable analyses comparing demographic, clinical and management characteristics between epidemic waves are summarised in Table 2. There was no change in gender or overall age distribution of patients between epidemic waves. After adjusting for other characteristics, patients admitted during wave 2 were less likely to be resident in an area with lower socio‐economic status, have a name consistent with an ethnic minority group, or have asthma, diabetes or neurological disease. During wave 2, patients were also less likely to receive antiviral therapy in the 2 days after onset, and less likely to be admitted in the 4 days after illness onset (Table 2).

**TABLE 2:** Association Between Characteristics of Hospitalised Patients with Influenza A(H1N1)pdm09 Virus Infections and Admission to Intensive Care—England, 2009‐2010

	Unadjusted odds ratio for ICU admission^a^ (95% Confidence Interval)	Adjusted odds ratio for ICU admission^a^ (95% Confidence Interval)	Unadjusted odds ratio for death^a^ (95% Confidence Interval)	Adjusted odds ratio for death^a^ (95% Confidence Interval)
Wave of hospitalisation^b^				
Wave 1	1	1	1	1
Wave 2	1.88 (1.36‐2.60)	1.88 (1.35‐2.61)^c^	1.80 (1.08‐3.00)	1.69 (1.00‐2.83)^c^
Region				
London/WM	1	1	1	1
Other regions	1.39 (1.01‐1.92)	1.22 (0.86‐1.73)^c^	1.24 (0.76‐2.02)	1.11 (0.65‐1.88)^c^
IMD Score				
Increased deprivation quartile	0.92 (0.80‐1.05)	0.98 (0.85‐1.13)^c^	0.99 (0.80‐1.22)	1.09 (0.87‐1.37)^c^
Ethnic Minority	0.61 (0.43‐0.87)	0.85 (0.58‐1.25)^c^	0.76 (0.45‐1.31)	1.19 (0.67‐2.12)^c^
Age on admission				
<15	1	1	1	1
15‐44	3.36 (2.19‐5.15)	3.40 (2.21‐5.24)^c^	4.39 (1.92‐10.04)	4.95 (2.16‐11.36)^c^
≥45	6.68 (4.29‐10.41)	6.67 (4.27‐10.41)^c^	13.02 (5.77‐29.35)	13.61 (6.02‐30.75)^c^
Male	0.91 (0.68‐1.22)	0.98 (0.73‐1.33)^c^	1.99 (1.23‐3.20)	2.17 (1.33‐3.52)^c^
Comorbidity				
Cardiac disease	3.10 (1.87‐5.13)	1.72 (0.97‐3.04)^d^	3.19 (1.55‐6.60)	1.22 (0.53‐2.81)^e^
Liver disease	1.97 (0.57‐6.78)	1.38 (0.37‐5.17)^d^	5.53 (1.59‐19.28)	4.71 (1.21‐18.27)^e^
Neurological disease	1.97 (1.17‐3.32)	2.74 (1.55‐4.83)^d^	2.56 (1.24‐5.26)	3.47 (1.58‐7.63)^e^
Renal disease	4.02 (2.23‐7.23)	2.70 (1.42‐5.12)^d^	5.60 (2.65‐11.81)	2.49 (1.07‐5.78)^e^
Asthma	0.64 (0.42‐0.96)	0.57 (0.37‐0.88)^d^	0.39 (0.18‐0.86)	0.37 (0.16‐0.82)^e^
Other respiratory disease	2.43 (1.56‐3.78)	1.24 (0.75‐2.07)^d^	2.43 (1.26‐4.70)	0.85 (0.40‐1.82)^e^
Diabetes	1.26 (0.62‐2.55)	0.58 (0.26‐1.30)^d^	2.29 (0.97‐5.43)	1.10 (0.41‐2.96)^e^
Immunocompromised	1.79 (1.08‐2.97)	1.16 (0.66‐2.03)^d^	4.22 (2.33‐7.62)	2.62 (1.35‐5.08)^e^
Obesity	10.09 (5.83‐17.48)	8.03 (4.49‐14.39)^d^	8.50 (4.20‐17.17)	6.58 (3.03‐14.30)^e^
Any specified comorbidity	2.28 (1.70‐3.08)	1.80 (1.32‐2.46)^c^	2.67 (1.66‐4.30)	1.81 (1.10‐2.97)^c^
Pregnancy	1.24 (0.70‐2.19)	1.18 (0.63‐2.23)^d^	0.42 (0.10‐1.73)	0.72 (0.16‐3.23)^e^
Onset to admission				
<2 d	1	1	1	1
2‐4 d	0.85 (0.60‐1.21)	0.71 (0.48‐1.03)^d^	1.17 (0.67‐2.04)	1.11 (0.62‐2.01)^e^
>4 d	1.22 (0.79‐1.88)	0.89 (0.55‐1.43)^d^	2.33 (1.28‐4.24)	1.91 (0.99‐3.68)^e^
Antivirals within 2 d^f^	0.80 (0.52‐1.22)	0.88 (0.56‐1.37)^d^	0.76 (0.43‐1.35)	0.96 (0.50‐1.84)^e^

Note

IMD: Index of Multiple Deprivation Score, by quartile of scores for the population of England.

^a^ ICU: intensive care unit; death refers to death from any cause in the 28 days after hospital admission.

^b^ Wave 1: 27th May 2009‐29th August 2009; Wave 2: 30th August 2009‐3rd January 2010.

^c^ Adjusted for age, sex, epidemic wave.

^d^ Adjusted for age, sex, epidemic wave, obesity, neurological disease, asthma, cardiac disease, renal disease.

^e^ Adjusted for age, sex, epidemic wave, obesity, neurological disease, asthma, liver disease, immunocompromise, renal disease.

^f^ For analysis of ICU admission, excludes antivirals received after admission to intensive care.

During wave 1, 54 patients (5.6%) were admitted to ICU, compared with 143 patients (10.1%) during wave 2; the odds ratio for admission to ICU during wave 2 compared with wave 1 was 1.88 (95% confidence interval [CI] 1.36‐2.60). Patients admitted during wave 2 had similarly increased odds of death within 28 days (1.80, CI 1.08‐3.00); there were 55 deaths (3.9% patients) during wave 2 compared with 21 deaths (2.2% patients) during wave 1. Differences in demographic, clinical and management characteristics among patients who were admitted to ICU or died are summarised in Table S1.

Since there was no significant evidence of interaction effects between wave and other covariates for the prediction of ICU and death, we used combined models to assess risk factors for these outcomes (Table 2). Compared with other patients, those admitted to ICU had increased odds of being in the age groups 15‐44 years (OR 3.36, CI 2.19‐5.15), or ≥45 years (OR 6.68, CI 4.29‐10.41). Patients who died had increased odds of being in the 15‐44 years age category (OR 4.39, CI 1.92‐10.04) or the ≥45 years age category (OR 13.02, CI 5.77‐29.35), and were more likely to be male (OR 1.99, 1.23‐3.20). Patients admitted to ICU were less likely to be from an ethnic minority group (OR 0.61, CI 0.43‐0.87), but we did not find other statistical associations between ethnic group or deprivation score and ICU admission or death. Patients with any previous comorbidity were more likely to be admitted to ICU (OR 2.28, 1.70‐3.08) or to die following the admission (OR 2.67, 1.66‐4.30); several specific comorbidities were also associated with severe outcomes (Table 2). Patients who died were more likely to have been admitted 4 or more days after symptom onset (OR 2.33, CI 1.28‐4.24). However, there was a weaker association between admission timing and ICU, and receiving antiviral therapy within 2 days after onset was not clearly associated with either measure of case‐severity.

Multivariable models of case‐severity are summarised in Table 2. We found that admission to ICU and death were both predicted by admission during wave 2, older age, neurological disease, renal disease and obesity, whereas admission with a diagnosis of asthma was associated with decreased odds for both outcomes. Death was also predicted by male gender, liver disease and immunocompromised status. Of factors predictive of increased case‐severity, only hospital admission outside London and the West Midlands and admission without asthma were statistically more likely during wave 2. After adjusting for other factors, patients admitted during wave 2 remained at increased odds of ICU admission (OR 1.88, CI 1.35‐2.61) and death (OR 1.69, CI 1.00‐2.83).

Results were similar after using multiple imputation to estimate the values of missing data. Missing data are summarised in relation to key analysis variables in Table S2, and imputed values are given in Table S3. Regression analyses for differences by wave using multiple imputation were similar to the primary analysis, except that admission during wave 2 was not significantly associated with decreased odds of asthma or overall comorbidity, whereas there was a statistical increase in the proportion of patients who were pregnant during wave 2 (Table S4). Analyses of risk factors for ICU and death were also similar using multiple imputation (Table S5).

## 4 DISCUSSION

We found that from the first to second epidemic waves there was an approximate two‐fold increase in the risk of severe outcomes of ICU admission and death among hospitalised patients. This is consistent with reports from previous studies^3^, ^9^ and included an increase in deaths during the 28 days after hospital admission. The increased case‐severity was accompanied by changes in populations affected, as well as in patient characteristics. However, we did not find that these reported changes could explain the observed increase in severity of hospitalised cases.

As transmission shifted between waves from London and the West Midlands to other regions, the subgroups most affected also changed. We found that a substantially higher proportion of patients admitted during the first wave were likely to be from ethnic minority groups, consistent with previous studies.^3^, ^5^ We also found an independent association with increased social deprivation score—more than 80% of patients admitted during the first wave were living in area with an above‐average score. However, the changes in ethnicity and deprivation score could not explain increased case‐severity, since neither attribute was associated with ICU admission or death. Early in the COVID‐19 pandemic, severe infections have also been associated with ethnic minority groups and patients with higher deprivation score.^19^, ^20^ Initial evidence has suggested that, similar to our findings, increased mortality from COVID‐19 among ethnic minority groups might have initially resulted from an increased risk of transmission, distinct from the risk for severe illness once hospitalised.^21^

Since older age is associated with ICU admission and death, a shift towards older ages during the second epidemic wave could theoretically explain the shift towards increased case‐severity. This has been noted in more recent influenza seasons because of greater immunity in children,^22^, ^23^ and other studies of pH1N1 during 2009/2010 found that patients were slightly older during the second wave.^3^, ^4^, ^5^, ^24^ However, we did not find a significant shift to older ages, possibly because the two waves occurred mostly in different regions. It is unclear why male patients had an increased risk of death but not ICU admission in our study, but the association appears consistent with a pooled analysis of pH1N1 severity, as well as more recent investigation of patients with COVID‐19.^19^, ^25^, ^26^, ^27^ Nevertheless, gender could not explain the change in severity because it did not differ between waves.

The increased risk of severe outcomes among patients with comorbidities is consistent with previous studies,^25^ but overall comorbidity was reported less frequently during the second wave. Of specific comorbidities predictive of severe outcomes, only obesity was reported more frequently during the second wave—although this difference was not statistically significant, and obesity was only reported for 21 ICU admissions and 9 deaths during the second wave. Only hospitalisation without a diagnosis of asthma was associated with the second wave and severe outcomes. The increased odds of ICU admission or death during the second wave were similar after adjusting for asthma diagnosis and other comorbidities, implying that none of these factors could account for the increased case‐severity during the second wave.

Similarly, we did not find evidence that changes in patient management between epidemic waves led to an increase in case‐severity. Consistent with other reports in England,^2^, ^3^ we found that antiviral use in the 2 days after illness onset was less frequent during the second wave, whereas hospital admission 4 or more days after admission was more frequent. However, neither factor predicted ICU admission or death after adjustment for other characteristics, and the association of admission during the second wave nor increased case‐severity persisted after adjustment for antiviral use and admission timing. Vaccination is unlikely to have led to increased severity of hospitalised cases since it might decrease rather than increase case‐severity, and by the end of the second wave, coverage remained less than 50% even in prioritised groups.^2^ Overall, we found that the increased odds of ICU admission and death during the second wave could not be explained by changes in any reported patient characteristics.

Before considering implications of our analysis, several limitations should be acknowledged. A high proportion of values were missing for some variables. Although we did not find substantial differences between the primary analysis and a secondary sensitivity analysis using multiple imputation, the accuracy of imputed values depends on an untestable assumption that values were “missing at random,” and predicted by other variables.^16^ It is also possible that severity changed because of changes in cases included in surveillance. A Bayesian analysis using the same dataset estimated that only 30% of all hospitalisations were included in the first wave, and 20% in the second wave.^7^ Change in admission practices could also have led to increased case‐severity; this possibility is suggested by intense media interest, higher consultation rates during the first wave, and a potential tendency to admit milder adult cases earlier in the pandemic.^9^ Our finding that patients with asthma had milder illness and were also more likely to be admitted during the first wave may be consistent with this, although this factor alone could not explain the increase in case‐severity. The slight increase in admissions delayed beyond 4 days in this study and in a previous study from sentinel surveillance^3^ could also reflect decisions to admit cases perceived to be more severe during the second wave. Nevertheless, the increased severity of hospitalised cases likely reflected a genuine increase in case‐severity among all infections,^9^ and we did not find evidence that changes in the types of patients admitted could explain more severe illness during the second wave.

Our study suggests that other unrecorded factors played an important role in the increased severity of hospitalised cases. Although the virus itself showed no genetic changes,^2^ seasonal changes in humidity and temperature have been found to drive changes in transmission^28^ and might increase clinical severity.^29^ In addition, the winter season is associated with circulation of a range of other viral and bacterial respiratory pathogens, which might increase risk of secondary infection and hence adverse outcomes.^30^ Autopsies of cases of pH1N1 indicated an important role of bacterial coinfections during 2009/2010, including from *S. pneumoniae*.^31^ Influenza coinfection is associated with a substantial component of invasive pneumococcal disease, which is also most frequent during winter months.^32^

This study demonstrates an increase in case‐severity between the summer and the autumn/winter waves during the 2009 influenza pandemic, highlighting the importance of real‐time surveillance systems to monitor severity and risk factors for severe disease across multiple epidemic waves. Our findings have several implications for responses to COVID‐19 as well as future influenza pandemics. First, the early increase in infections among ethnic minorities and groups of lower socio‐economic status indicates that it is important to identify demographic groups at elevated risk of infection to prevent further transmission and morbidity. Second, changes in the severity of hospitalised cases need to be monitored to support prioritisation of healthcare resources. Third, reasons for changes in severity need to be understood in order to predict changes in severe outcomes and to ensure that vaccines, therapeutics and other public health interventions can be targeted most effectively. To explain changes in case‐severity over time, surveillance systems will need to account for changes in thresholds for admission and seasonal factors such as bacterial coinfection, in addition to changes in other patient characteristics.

## CONFLICT OF INTEREST

All authors have no conflicts of interest to declare.

## AUTHOR CONTRIBUTIONS

**Ian D. Plumb:** Conceptualization (equal); Formal analysis (equal); Writing‐original draft (lead). **Ross Harris:** Data curation (supporting); Formal analysis (equal); Writing‐review & editing (equal). **Helen K. Green:** Data curation (lead); Formal analysis (equal); Writing‐review & editing (equal). **Joanna Ellis:** Resources (lead); Writing‐review & editing (equal). **Kathy Baisley:** Conceptualization (supporting); Supervision (supporting); Writing‐review & editing (equal). **Richard** **Pebody:** Conceptualization (lead); Supervision (lead); Writing‐review & editing (lead).

## ETHICS AND CONSENT

Study data were collected as part of routine pandemic surveillance under the NHS Act 2006 (section 251); explicit ethical approval and individual patient consent were not required.

### PEER REVIEW

The peer review history for this article is available at https://publons.com/publon/10.1111/irv.12863.

## Supporting information

Table S1‐S5

## Data Availability

Study data are not shared.
