# Supplementary material for: Changes in characteristics and case‐severity in patients hospitalised with influenza A (H1N1) pdm09 infection between two epidemic waves—England, 2009–2010
Source: Influenza Other Respir Viruses. 2021 May 4;15(5):599–607. doi: 10.1111/irv.12863 (PMC8404053; doi:10.1111/irv.12863)
Supplement: Supplementary file 1 — Table S1‐S5 [file IRV-15-599-s001.docx]

Supplementary Table 1: Differences in Characteristics of Hospitalised Patients with Influenza A(H1N1)pdm09 Virus Infections Between Two Epidemic Waves — England, 2009–2010

|  | **Admissions to ICU** | | **Deaths within 28 days ^†^** | |
| --- | --- | --- | --- | --- |
|  | **No. in wave 1/ all cases (%)** | **No. in wave 2/ all cases (%)** | **No. in wave 1/ all cases (%)** | **No. in wave 2/ all cases (%)** |
| **Region** |  |  |  |  |
| London/WM | 30/54 (56%) | 28/143 (20%) | 11/21 (52%) | 13/55 (24%) |
| Other regions | 24/54 (44%) | 115/143 (80%) | 10/21 (48%) | 42/55 (76%) |
| **IMD Quartiles ‡** |  |  |  |  |
| Least deprived | 11/50 (22%) | 54/125 (43%) | 3/18 (17%) | 21/51 (41%) |
| Most deprived | 39/50 (78%) | 71/125 (57%) | 15/18 (83%) | 30/51 (59%) |
| **Ethnicity** |  |  |  |  |
| White | 35/54 (65%) | 122/143 (85%) | 11/21 (52%) | 47/55 (85%) |
| Minority | 19/54 (35%) | 21/143 (15%) | 10/21 (48%) | 8/55 (15%) |
| **Age (years)** |  |  |  |  |
| <15 | 8/54 (19%) | 21/143 (15%) | 3/21 (14%) | 4/55 (7%) |
| 15–44 | 29/54 (54%) | 62/143 (43%) | 10/21 (48%) | 20/55 (36%) |
| ≥45 | 17/54 (31%) | 60/143 (42%) | 8/21 (38%) | 31/55 (56%) |
| **Gender** |  |  |  |  |
| Male | 24/54 (44%) | 67/143 (47%) | 12/21 (57%) | 37/55 (67%) |
| Female | 30/54 (56%) | 76/143 (53%) | 9/21 (43%) | 18/55 (33%) |
| **Comorbidity** |  |  |  |  |
| Cardiac disease | 6/54 (11%) | 15/143 (10%) | 2/21 (10%) | 7/55 (13%) |
| Liver disease | 0/54 (0%) | 3/143 (2%) | 1/21 (5%) | 2/55 (4%) |
| Neurological disease | 6/54 (11%) | 12/143 (8%) | 2/21 (10%) | 7/55 (13%) |
| Renal disease | 5/54 (9%) | 11/143 (8%) | 2/21 (10%) | 7/55 (13%) |
| Asthma | 8/54 (15%) | 20/143 (14%) | 4/21 (19%) | 3/55 (5%) |
| Other respiratory disease | 9/54 (17%) | 18/143 (13%) | 3/21 (14%) | 8/55 (15%) |
| Diabetes | 3/54 (6%) | 6/143 (4%) | 4/21 (19%) | 2/55 (4%) |
| Immunocompromised | 6/54 (11%) | 13/143 (9%) | 4/21 (19%) | 11/55 (20%) |
| Obesity | 4/54 (7%) | 21/143 (15%) | 2/21 (10%) | 9/55 (16%) |
| Any specified comorbidity | 31/54 (57%) | 87/143 (61%) | 13/21 (62%) | 36/55 (65%) |
| Pregnancy | 3/54 (6%) | 11/143 (8%) | 2/21 (10%) | 0/55 (0%) |
| **Onset to admission** |  |  |  |  |
| <2 days | 21/49 (43%) | 85/136 (63%) | 6/18 (33%) | 27/53 (51%) |
| 2–4 days | 23/49 (47%) | 27/136 (20%) | 9/18 (50%) | 12/53 (23%) |
| >4 days | 5/49 (10%) | 24/136 (18%) | 3/18 (17%) | 14/53 (26%) |
| **Antivirals within 2 days §** | 13/40 (33%) | 24/100 (24%) | 7/20 (35%) | 10/37 (27%) |
| **Intensive care admission** | - | - | 11/21 (52%) | 40/55 (73%) |
| **Death ≤28 days** | 11/54 (20%) | 40/143 (28%) | - | - |

† Deaths defined as occurring within 28 days of admission, from any cause. ‡ IMD: Index of Multiple Deprivation Score, by quartile of scores for the population of England. § Excluding if received antivirals after admission to intensive care

Supplementary Table 2: Distributions of observed and missing values in relation to key analysis variables

|  |  | **Admitted in second wave (No. /**  **all patients [%])** | **Admitted to intensive care unit (No. /**  **all patients [%])** | **Died within 28 days after admission (No. /**  **all patients [%])** |
| --- | --- | --- | --- | --- |
| All cases |  | 1420/2380 (60%) | 197/2380 (8%) | 76/2380 (3%) |
| ICU admission | Complete | 880/1471 (60%) | - | 65/1471 (4%) |
|  | Missing | 540/909 (59%) | - | 11/909 (1%) |
|  | Not admitted to ICU | 737/1274 (58%) | - | 14/1274 (1%) |
|  |  |  |  |  |
| Antiviral use within 2 days | Complete | 989/1719 (58%) | 140/1719 (8%) | 57/1719 (3%) |
|  | Missing | 431/661 (65%) | 57/661 (9%) | 19/661 (3%) |
|  |  |  |  |  |
| Onset to admission | Complete | 1419/2312 (61%) | 193/2312 (8%) | 73/2312 (3%) |
|  | Missing | 1/68 (1%) | 4/68 (6%) | 3/68 (4%) |
|  |  |  |  |  |
| Deprivation score | Complete | 1285/2110 (61%) | 175/2110 (8%) | 69/2110 (3%) |
|  | Missing | 135/270 (50%) | 22/270 (8%) | 7/270 (3%) |
|  |  |  |  |  |
| Comorbidity | Complete | 871/1542 (56%) | 143/1542 (9%) | 57/1542 (4%) |
|  | Missing | 549/838 (66%) | 54/838 (6%) | 19/838 (2%) |
|  | No comorbidity | 317/561 (57%) | 25/561 (4%) | 8/561 (1%) |
|  |  |  |  |  |
| Duration of admission | Complete | 1288/2105 (61%) | 97/2105 (5%) | 6/2105 (0%) |
|  | Missing | 132/275 (48%) | 100/275 (36%) | 70/275 (25%) |

Supplementary Table 3 – Comparison of observed and imputed values for different variables

| **Characteristic** | **% (number) in observed data** | **% (number) in**  **imputed data** |
| --- | --- | --- |
| Age <15 years | 40.6% (2380) | N/A (0) |
| Admitted in West Midlands or London | 63.8% (2380) | N/A (0) |
| Female sex | 51.7% (2375) | 56.2% (5) |
| Lowest quartile of index of deprivation | 16.3% (2110) | 12.0% (270) |
| Heart disease | 5.0% (2041) | 7.0% (339) |
| Liver disease | 1.0% (2040) | 2.0% (340) |
| Neurological disease | 6.0% (2051) | 7.2% (329) |
| Renal disease | 3.1% (2047) | 4.6% (333) |
| Respiratory disease | 7.7% (2078) | 0.0% (302) |
| Diabetes | 4.4% (2045) | 5.0% (335) |
| Immunocompromised | 7.0% (2031) | 9.1% (349) |
| Obesity | 5.0% (1118) | 8.7% (1262) |
| Any comorbidity | 63.6% (1542) | 55.3% (838) |
| Pregnancy | 9.0% (1574) | 7.9% (806) |
| Antivirals ≤ 2 days (if pre-ICU) | 36.2% (1684) | 40.2% (696) |
| Admission < 2 days post onset | 56.3% (2242) | 47.4% (138) |
| Intensive care admission | 13.4% (1471) | 8.3% (909) |
| Ventilation | 7.8% (2000) | 8.6% (380) |

Supplementary Table 4: Association Between Characteristics of Hospitalised Patients with Influenza A(H1N1)pdm09 Virus Infections and Epidemic Wave of Admission During Wave 2 — England, 2009–2010 (using imputed data)

|  | **No. in wave 1 /**  **All cases (%)** | **No. in wave 2 /**  **All cases (%)** | **Unadjusted OR † (95% confidence interval)** | **Adjusted OR †**  **(95% confidence interval)** |
| --- | --- | --- | --- | --- |
| **Region** |  |  |  |  |
| London/WM | 576/960 (60.0%) | 285/1420 (20.1%) | 1 (ref) | 1 (ref) |
| Other regions | 384/960 (40.0%) | 1135/1420 (79.9%) | 5.97 (4.97-7.17) | 4.22 (3.47-5.13) ^§^ |
| **IMD Score ‡** |  |  |  |  |
| Less deprived | 182/960 (18.9%) | 599/1420 (42.2%) | 1 (ref) | 1 (ref) |
| More deprived | 778/960 (81.1%) | 821/1420 (57.8%) | 0.32 (0.26-0.39) | 0.54 (0.43-0.67) ^§^ |
| **Ethnicity** |  |  |  |  |
| White | 503/960 (52.4%) | 1193/1420 (84.0%) | 1 (ref) | 1 (ref) |
| Minority/other | 457/960 (47.6%) | 227/1420 (16.0%) | 0.21 (0.17-0.25) | 0.34 (0.28-0.42) ^§^ |
| **Age on admission (years)** |  |  |  |  |
| <15 | 390/960 (40.6%) | 575/1420 (40.5%) | 1 (ref) | 1 (ref) |
| 15 to 44 | 403/960 (42.0%) | 563/1420 (39.6%) | 0.95 (0.79-1.14) | 1.00 (0.81-1.23) ^§^ |
| ≥45 | 167/960 (17.4%) | 282/1420 (19.9%) | 1.15 (0.91-1.44) | 1.08 (0.83-1.40) ^§^ |
| **Gender** |  |  |  |  |
| Female | 503/960 (52.4%) | 727/1420 (51.2%) | 1 (ref) | 1 (ref) |
| Male | 457/960 (47.6%) | 693/1420 (48.8%) | 1.05 (0.89-1.24) | 1.00 (0.83-1.21) ^§^ |
| **Comorbidity** |  |  |  |  |
| Cardiac disease | 53/960 (5.5%) | 73/1420 (5.2%) | 0.94 (0.64-1.37) | 0.94 (0.60-1.49) ^¶^ |
| Liver disease | 8/960 (0.9%) | 18/1420 (1.3%) | 1.50 (0.60-3.72) | 2.39 (0.85-6.71) ^¶^ |
| Neurological disease | 74/960 (7.7%) | 76/1420 (5.3%) | 0.68 (0.47-0.97) | 0.63 (0.42-0.95) ^¶^ |
| Renal disease | 28/960 (2.9%) | 51/1420 (3.6%) | 1.23 (0.74-2.05) | 1.45 (0.80-2.64) ^¶^ |
| Asthma | 231/960 (24.0%) | 320/1420 (22.5%) | 0.92 (0.75-1.13) | 0.81 (0.64-1.02) ^¶^ |
| Other respiratory disease | 107/960 (11.1%) | 139/1420 (9.8%) | 0.87 (0.66-1.15) | 0.80 (0.58-1.12) ^¶^ |
| Diabetes | 62/960 (6.5%) | 44/1420 (3.1%) | 0.46 (0.30-0.71) | 0.40 (0.24-0.67) ^¶^ |
| Immunocompromised | 64/960 (6.7%) | 112/1420 (7.9%) | 1.19 (0.86-1.66) | 1.12 (0.76-1.65) ^¶^ |
| Obesity | 50/960 (5.2%) | 117/1420 (8.2%) | 1.63 (0.95-2.80) | 1.33 (0.69-2.57) ^¶^ |
| Any specified comorbidity | 597/960 (62.2%) | 872/1420 (61.4%) | 0.96 (0.79-1.18) | 0.86 (0.67-1.11) ^¶^ |
| Pregnancy | 72/960 (7.5%) | 141/1420 (9.9%) | 1.37 (0.98-1.90) | 2.39 (1.52-3.74) ^¶^ |
| **Onset to admission** |  |  |  |  |
| <2 days | 507/960 (52.8%) | 823/1420 (58.0%) | 1 (ref) | 1 (ref) |
| 2 to 4 days | 334/960 (34.8%) | 403/1420 (28.4%) | 0.74 (0.61-0.91) | 0.71 (0.57-0.89) ^¶^ |
| >4 days | 120/960 (12.5%) | 193/1420 (13.6%) | 1.00 (0.76-1.30) | 0.84 (0.62-1.15) ^¶^ |
| **Antivirals within 2 days ††** | 414/960 (43.2%) | 487/1420 (34.3%) | 0.69 (0.56-0.85) | 0.73 (0.58-0.91) ^¶^ |
| **Intensive care admission** | 78/960 (8.1%) | 186/1420 (13.1%) | 1.71 (1.22-2.40) | 1.58 (1.05-2.37) ^¶^ |
| **Death <28 days** | 21/960 (2.2%) | 55/1420 (3.9%) | 1.80 (1.08-3.00) | 1.89 (1.04-3.44) ^¶^ |

† Odds ratios (ORs) for hospital admission during wave 1 (before 30^th^ August 2009) compared with admission during wave 2 (on or after 30^th^ August 2009) as baseline ‡ IMD: Index of Multiple Deprivation Score, by quartile of scores for the population of England. § Adjusted for region, IMD quartile, ethnic group, age, sex ¶ Adjusted for region, IMD quartile, ethnic group, age, sex, neurological disease, diabetes, asthma. †† Excluding if received antivirals after admission to intensive care

Supplementary Table 5 – Association Between Characteristics of Hospitalised Patients with Influenza A(H1N1)pdm09 Virus Infections and Admission to ICU or Death within 28 days— England, 2009–2010 (using imputed data)

|  | **Association of factors with ICU admission** | | **Association of factors with death** | |
| --- | --- | --- | --- | --- |
|  | **Unadjusted OR †** | **Adjusted OR †** | **Unadjusted OR †** | **Adjusted OR †** |
|  | **(95% Confidence Interval)** | **(95% Confidence Interval)** | **(95% Confidence Interval)** | **(95% Confidence Interval)** |
| **Wave of hospitalisation ^‡^** |  |  |  |  |
| Wave 1 | 1 (ref) | 1 (ref) | 1 (ref) | 1 (ref) |
| Wave 2 | 1.71 (1.22-2.40) | 1.55 (1.05-2.29) ^¶^ | 1.80 (1.08-3.00) | 1.70 (0.97-2.98) ^¶^ |
| **Region** |  |  |  |  |
| London/WM | 1 (ref) | 1 (ref) | 1 (ref) | 1 (ref) |
| Other regions | 1.38 (1.01-1.89) | 1.26 (0.87-1.82) ^¶^ | 1.24 (0.76-2.02) | 1.20 (0.69-2.08) ^¶^ |
| **IMD Score ^¶^** |  |  |  |  |
| Least deprived | 1 (ref) | 1 (ref) | 1 (ref) | 1 (ref) |
| Most deprived | 0.89 (0.65-1.21) | 1.07 (0.77-1.50) ^¶^ | 0.92 (0.56-1.51) | 1.11 (0.66-1.89) ^¶^ |
| **Ethnicity** |  |  |  |  |
| White | 1 (ref) | 1 (ref) | 1 (ref) | 1 (ref) |
| Minority/other | 0.64 (0.45-0.92) | 0.91 (0.59-1.38) ^¶^ | 0.76 (0.45-1.31) | 1.22 (0.67-2.22) ^¶^ |
| **Age on admission (years)** |  |  |  |  |
| <15 | 1 (ref) | 1 (ref) | 1 (ref) | 1 (ref) |
| 15 to 44 | 3.44 (2.33-5.07) | 3.50 (2.37-5.18) ^¶^ | 4.39 (1.92-10.04) | 5.00 (2.18-11.49) ^¶^ |
| ≥45 | 6.40 (4.21-9.74) | 6.43 (4.21-9.84) ^¶^ | 13.02 (5.77-29.35) | 14.07 (6.19-31.97) ^¶^ |
| **Gender** |  |  |  |  |
| Female | 1 (ref) | 1 (ref) | 1 (ref) | 1 (ref) |
| Male | 0.90 (0.67-1.22) | 0.98 (0.72-1.34) ^¶^ | 1.99 (1.23-3.20) | 2.18 (1.34-3.55) ^¶^ |
| **Comorbidity** |  |  |  |  |
| Cardiac disease | 3.27 (2.00-5.33) | 2.18 (1.25-3.79) †† | 3.99 (1.97-8.08) | 1.78 (0.81-3.91) ‡‡ |
| Liver disease | 2.51 (0.77-8.20) | 2.17 (0.64-7.31) †† | 6.50 (1.88-22.45) | 5.97 (1.50-23.76) ‡‡ |
| Neurological disease | 2.27 (1.41-3.65) | 3.22 (1.90-5.46) †† | 2.88 (1.44-5.75) | 3.77 (1.75-8.16) ‡‡ |
| Renal disease | 5.28 (2.81-9.89) | 4.12 (2.09-8.11) †† | 6.39 (2.99-13.68) | 3.74 (1.61-8.65) ‡‡ |
| Asthma | 0.86 (0.58-1.29) | 0.70 (0.47-1.06) †† | 0.47 (0.22-1.00) | 0.39 (0.18-0.84) ‡‡ |
| Other respiratory disease | 2.29 (1.51-3.49) | 1.56 (0.97-2.50) †† | 2.67 (1.46-4.88) | 1.46 (0.76-2.83) ‡‡ |
| Diabetes | 1.46 (0.76-2.80) | 0.98 (0.49-1.97) †† | 2.49 (1.05-5.93) | 1.36 (0.53-3.46) ‡‡ |
| Immunocompromised | 2.14 (1.34-3.41) | 1.63 (0.98-2.71) †† | 4.92 (2.66-9.08) | 3.53 (1.79-6.95) ‡‡ |
| Obesity | 6.55 (3.65-11.76) | 5.24 (2.87-9.59) †† | 5.94 (3.02-11.66) | 4.48 (2.14-9.38) ‡‡ |
| Any specified comorbidity | 3.72 (2.31-5.99) | 3.06 (1.82-5.14) ^¶^ | 3.62 (1.69-7.76) | 2.41 (1.02-5.70) ^¶^ |
| Pregnancy | 1.31 (0.75-2.29) | 1.08 (0.55-2.14) †† | 0.45 (0.13-1.61) | 0.59 (0.15-2.41) ‡‡ |
| **Onset to admission** |  |  |  |  |
| <2 days | 1 (ref) | 1 (ref) | 1 (ref) | 1 (ref) |
| 2 to 4 days | 0.72 (0.51-1.00) | 0.66 (0.46-0.93) †† | 1.17 (0.67-2.04) | 1.14 (0.64-2.03) ‡‡ |
| >4 days | 1.07 (0.69-1.67) | 0.93 (0.58-1.48) †† | 2.28 (1.26-4.15) | 2.14 (1.15-3.99) ‡‡ |
| **Antivirals within 2 days** §§ | 0.80 (0.53-1.20) | 0.83 (0.55-1.27) †† | 0.80 (0.46-1.39) | 0.88 (0.50-1.54) ‡‡ |

†† Odds ratios (ORs) for admission to ICU compared with hospital admission without ICU admission calculated using logistic regression ‡ Wave 1 was defined as 27th May 2009 to 30th August 2009, and wave 2 from 30th August 2009 to 3rd January 2010. § IMD: Index of Multiple Deprivation Score, by quartile of scores for the population of England. § Adjusted for age, sex, epidemic wave ¶ Adjusted for age, sex, epidemic wave, obesity, neurological disease, asthma, cardiac disease, renal disease. ‡‡ Adjusted for age, sex, epidemic wave, obesity, neurological disease, asthma, liver disease, immunocompromised, renal disease §§ Excluding if received antivirals after admission to intensive care
